# A New Era of Treatment Sequencing in Platinum-Resistant Ovarian Cancer: Integrating Immunotherapy, Antibody–Drug Conjugates, and Targeted Therapies

**DOI:** 10.3390/curroncol33100622

**Published:** 2026-10-15

**Authors:** Seiji Mabuchi, Ai Kusakabe, Yumi Takimoto

**Affiliations:** Department of Obstetrics and Gynecology, Hyogo Medical University, 1-1, Mukogawa-Cho, Nishinomiya 663-8501, Japan; ai-kusakabe@hyo-med.ac.jp (A.K.); yu-takimoto@hyo-med.ac.jp (Y.T.)

**Keywords:** platinum-resistant ovarian cancer, treatment sequencing, immunotherapy, antibody–drug conjugates, targeted therapy, biomarkers

## Abstract

Platinum-resistant ovarian cancer (PROC) has historically been treated with sequential non-platinum chemotherapy, with bevacizumab added when appropriate. The therapeutic landscape has expanded with positive randomized phase III data for pembrolizumab plus weekly paclitaxel with or without bevacizumab (KEYNOTE-B96), mirvetuximab soravtansine (MIRV) in FRα-high disease (MIRASOL), and relacorilant plus nab-paclitaxel (ROSELLA). Trastuzumab deruxtecan (T-DXd) has also shown substantial activity in previously treated HER2-expressing ovarian cancer, although its ovarian cohort was small, nonrandomized, and not restricted to PROC. These trials enrolled different populations and do not establish a cross-trial efficacy hierarchy. We therefore review the development of immunotherapy leading to KEYNOTE-B96, compare newer strategies with established PROC treatments, distinguish phase III evidence from regulatory approval and routine availability, and propose an evidence-informed framework for treatment selection and sequencing. The framework prioritizes current treatment opportunity while accounting for biomarker eligibility, prior treatment, residual toxicity, treatment and financial burden, patient priorities, and the feasibility of later-line therapy.

## Simple Summary

Platinum-resistant ovarian cancer (PROC) is no longer managed only with sequential single-agent chemotherapy. Randomized phase III trials now support pembrolizumab plus weekly paclitaxel with or without bevacizumab, mirvetuximab soravtansine for FRα-high disease, and relacorilant plus nab-paclitaxel, while HER2-directed trastuzumab deruxtecan provides an additional biomarker-defined option in selected patients. This review places these newer treatments alongside established chemotherapy and bevacizumab-based regimens and proposes a clinical framework for choosing among them. The framework considers biomarker eligibility, prior therapy, residual toxicity, visit and monitoring requirements, financial accessibility, and the likelihood that a patient will remain fit for subsequent treatment.

## 1. Introduction

Platinum-resistant ovarian cancer (PROC) remains one of the most challenging settings in gynecologic oncology. For more than two decades, treatment has relied largely on sequential non-platinum chemotherapy, most commonly weekly paclitaxel, pegylated liposomal doxorubicin (PLD), topotecan, or gemcitabine, with bevacizumab added when appropriate [1,2,3,4]. Oral etoposide and metronomic oral cyclophosphamide, including cyclophosphamide-bevacizumab combinations, can also be considered in selected patients when standard intravenous options are unsuitable or have been exhausted, although their supporting evidence is less robust than that for the principal randomized strategies discussed below [5,6]. As a benchmark for conventional therapy, AURELIA reported a median PFS of 3.4 months and median OS of 13.3 months with investigator-choice single-agent chemotherapy; adding bevacizumab improved median PFS to 6.7 months, while the OS difference was not statistically significant [3].

Recent phase III studies have added three distinct strategies to the PROC evidence base: pembrolizumab plus weekly paclitaxel with or without bevacizumab in KEYNOTE-B96 [7], FRα-directed MIRV in MIRASOL [8,9], and relacorilant plus nab-paclitaxel in ROSELLA [10]. T-DXd adds a separate HER2-directed option, but the supporting ovarian-cancer data come from a nonrandomized phase II basket study rather than a PROC-specific randomized trial [11].

The resulting clinical problem is not simply to identify an active drug, but to choose among several treatments that differ in evidence level, biomarker requirements, toxicity, monitoring, access, and practical burden. Because some patients deteriorate before another line of therapy can be delivered, a potentially effective treatment should not be deferred solely to keep it available for later use. Sequencing decisions should instead balance the expected value of treatment now against residual toxicity and the realistic feasibility of subsequent therapy.

In this review, we examine the major evidence-supported strategies now relevant to PROC, including chemoimmunotherapy, FRα-directed therapy, glucocorticoid-receptor antagonism combined with nab-paclitaxel, HER2-directed ADC therapy, and established non-platinum regimens. We first place the phase III KEYNOTE-B96 findings in the context of earlier immunotherapy studies, then examine MIRV, relacorilant plus nab-paclitaxel, and T-DXd using the same domains of trial population, biomarker requirements, efficacy, toxicity, and treatment burden. Finally, we consider how these options might be integrated across successive lines of treatment and propose a practical framework for longitudinal treatment sequencing in PROC.

## 2. Materials and Methods

### Literature Search and Review Scope

This narrative review was based on searches of PubMed/MEDLINE, ClinicalTrials.gov, and major regulatory and guideline sources. Search terms included combinations of “platinum-resistant ovarian cancer,” “pembrolizumab,” “paclitaxel,” “KEYNOTE-B96,” “mirvetuximab soravtansine,” “MIRASOL,” “relacorilant,” “ROSELLA,” “trastuzumab deruxtecan,” “HER2,” “immune checkpoint inhibitor,” “treatment sequencing,” “bevacizumab,” “topotecan,” “pegylated liposomal doxorubicin,” “etoposide,” and “cyclophosphamide.”

We prioritized randomized phase II/III trials, pivotal prospective studies, primary translational studies, regulatory documents, and contemporary clinical-practice guidelines. Single-arm or retrospective studies were included when they addressed a clinically relevant question not covered by randomized evidence, such as post-MIRV treatment or selected later-line conventional options. Reports unrelated to ovarian cancer were excluded unless they provided mechanistic evidence directly relevant to a treatment discussed in this review.

The search covered database inception through 22 September 2026, which was the final literature and regulatory search date. Because this was a narrative rather than systematic review, study selection was based on relevance to the biological rationale, clinical evidence, practical implementation, and treatment-sequencing questions addressed in the manuscript; no formal meta-analysis or risk-of-bias assessment was performed.

## 3. Lessons from the Development of Immunotherapy in Platinum-Resistant Ovarian Cancer

### 3.1. Biological Rationale for Immunotherapy

Several observations provided the initial rationale for checkpoint inhibition in ovarian cancer. Higher densities of intratumoral or intraepithelial T cells, particularly CD8-positive TILs, have been associated with longer survival, whereas regulatory T cells and other suppressive elements correlate with less favorable outcomes [12,13,14,15]. MDSCs can further inhibit CD8-positive T-cell function and promote tumor PD-L1 expression, adding another route of immune escape [16].

The PD-1/PD-L1 pathway is active in a subset of ovarian cancers, but its expression is heterogeneous and depends on histology, assay, and scoring method. In high-grade serous disease, reported PD-L1 positivity differs substantially according to whether tumor proportion score or combined positive score is used [17]. At the same time, ovarian cancer generally carries a relatively low tumor mutational burden, which may limit the neoantigen pool available to support checkpoint-inhibitor monotherapy [18,19].

Taken together, these data support the presence of antitumor immune recognition but also show why checkpoint blockade alone may be inadequate. Ovarian tumors contain multiple suppressive components, including dysfunctional T cells, myeloid populations, abnormal vasculature, stromal barriers, and inhibitory cytokine or metabolic networks [15,18]. The clinical question was therefore not whether immune recognition existed, but whether PD-1/PD-L1 blockade by itself could produce enough disease control to alter outcomes.

### 3.2. ICI Monotherapy: Biological Activity, but Insufficient Clinical Benefit

Early trials answered this question relatively clearly (Table 1). In the phase Ib KEYNOTE-028 study, pembrolizumab produced an objective response rate (ORR) of 11.5%, with median progression-free survival (PFS) of 1.9 months and median overall survival (OS) of 13.8 months in patients with PD-L1-positive advanced ovarian cancer [20]. Although this established proof of concept for PD-1 blockade, the low response rate and short PFS indicated insufficient activity for most patients.

The larger phase II KEYNOTE-100 study of pembrolizumab monotherapy confirmed this limitation. The overall ORR was approximately 8% and median PFS was 2.1 months, although ORR increased with increasing PD-L1 combined positive score (CPS), suggesting modest enrichment for response [21]. Thus, PD-L1 CPS enriched for response but did not identify a population in which pembrolizumab monotherapy provided adequate disease control.

The phase III NINJA trial provided the definitive randomized test of ICI monotherapy in PROC. Nivolumab was compared with investigator’s-choice gemcitabine or pegylated liposomal doxorubicin (PLD). ORR was 7.6% versus 13.2%, median PFS 2.0 versus 3.8 months, and median OS 10.1 versus 12.1 months, respectively [22]. Nivolumab therefore failed to improve survival and provided inferior disease control compared with conventional chemotherapy.

The monotherapy experience was therefore consistent across studies: a small subset of patients could have durable responses, but response rates near 8–12% and median PFS around 2 months were not sufficient to displace chemotherapy in routine PROC care. Subsequent development consequently focused on combinations capable of increasing the depth or frequency of response.

### 3.3. Combination Strategies: Improved Activity Did Not Necessarily Establish a New Standard

Combination development then moved in several directions. TOPACIO/KEYNOTE-162 reported an ORR of 18%, disease control in 65%, and median PFS of 3.4 months with niraparib (a PARP inhibitor) plus pembrolizumab [23], but its single-arm design could not establish comparative benefit. Larger first-line programs later tested PARP inhibitor-ICI strategies: FIRST showed a modest PFS gain without an OS benefit, whereas DUO-O improved PFS with a durvalumab (an anti-PD-L1 antibody)/bevacizumab/olaparib (a PARP inhibitor) strategy whose design did not isolate the contribution of the PARP inhibitor-ICI interaction [26,27]. These results did not establish a PARP inhibitor-ICI standard for PROC.

The randomized phase II NRG-GY003 trial provided stronger evidence that increasing immune activation could improve antitumor activity. Nivolumab plus ipilimumab increased ORR from 12.2% to 31.4% and median PFS from 2.0 to 3.9 months compared with nivolumab alone; median OS was 28.1 versus 21.8 months, without a significant OS difference [24].

However, the phase III JAVELIN Ovarian 200 trial demonstrated that combining an ICI with chemotherapy was not sufficient by itself. Avelumab, an anti-PD-L1 monoclonal antibody, was evaluated as monotherapy and in combination with PLD. Median PFS was 1.9, 3.7, and 3.5 months and median OS was 11.8, 15.7, and 13.1 months with avelumab, avelumab plus PLD, and PLD alone, respectively; neither avelumab-containing strategy significantly improved PFS or OS [25].

These studies established that ICI monotherapy was insufficient and that combining an ICI with another active treatment did not, by itself, guarantee improvement in PFS or OS.

## 4. Chemoimmunotherapy in PROC: KEYNOTE-B96 in Context

### 4.1. Phase III Evidence from KEYNOTE-B96

KEYNOTE-B96 should be viewed in the context of these earlier immunotherapy trials rather than as an isolated positive study. Earlier studies established biological activity of immune checkpoint inhibition in ovarian cancer, but ICI monotherapy produced responses in only a minority of patients, and subsequent combination strategies had not demonstrated a phase III survival benefit. JAVELIN Ovarian 200 further showed that simply adding an ICI to chemotherapy was insufficient to improve clinical outcomes [20,21,22,23,24,25,26,27].

ENGOT-ov65/KEYNOTE-B96 changed this record by demonstrating phase III PFS and OS benefit for checkpoint inhibition in PROC [7]. Participants received pembrolizumab or placebo with weekly paclitaxel; bevacizumab was permitted according to investigator assessment and planned use was a stratification factor. Because bevacizumab itself was not randomized, the study establishes the incremental value of pembrolizumab on the paclitaxel ± bevacizumab backbone but cannot determine whether bevacizumab altered that incremental effect.

In the intention-to-treat (ITT) population, pembrolizumab-containing therapy improved median PFS from 6.4 to 8.3 months (HR 0.70, 95% CI 0.58–0.84; *p* < 0.0001), median OS from 14.0 to 17.7 months (HR 0.82, 95% CI 0.69–0.97; *p* = 0.011), and ORR from 40.8% to 50.4% [7]. The benefit was more pronounced in the prespecified PD-L1 CPS ≥ 1 population, in which median PFS was 8.3 versus 7.2 months (HR 0.72, 95% CI 0.58–0.89; *p* = 0.0014) and median OS was 18.2 versus 14.0 months (HR 0.76, 95% CI 0.61–0.94; *p* = 0.0053) [7].

The improved efficacy was accompanied by greater toxicity. Grade ≥ 3 treatment-related adverse events occurred in 68% of patients receiving pembrolizumab plus paclitaxel and 55% receiving placebo plus paclitaxel [7]. Common treatment-related adverse events included anemia, peripheral neuropathy, alopecia, fatigue, and nausea. Treatment-related deaths occurred in 1% and 2%, respectively; in the pembrolizumab group, reported causes included colitis and interstitial lung disease, illustrating the additional possibility of clinically important immune-mediated toxicity [7].

Thus, the importance of KEYNOTE-B96 lies not simply in demonstrating greater tumor response, but in converting the previously modest biological activity of checkpoint inhibition into improvements in both PFS and OS. Its clinical value must nevertheless be weighed against the added toxicity and treatment burden of chemoimmunotherapy.

### 4.2. Weekly Paclitaxel as the Chemotherapy Backbone

Weekly paclitaxel was an important design feature of KEYNOTE-B96. Although carboplatin and paclitaxel are commonly administered together in first-line therapy, platinum resistance does not necessarily imply complete resistance to paclitaxel. Indeed, weekly paclitaxel has demonstrated clinically meaningful activity even after previous taxane exposure and in tumors clinically resistant or refractory to both platinum and conventionally scheduled paclitaxel, with ORRs of approximately 20–25% in phase II studies [3,28,29]. Prior taxane sensitivity nevertheless remains relevant: in a randomized phase II study, response, PFS, and OS with weekly paclitaxel were significantly better in patients with a taxane-free interval ≥ 6 months or no prior taxane than in those with a shorter interval [30]. The independent antitumor activity of weekly paclitaxel is therefore an important part of the KEYNOTE-B96 design and should be considered separately from any hypothesized immunomodulatory contribution.

Taxanes may also have immunomodulatory effects. Preclinical studies suggest that paclitaxel can reduce suppressive myeloid populations, restore CD8-positive T-cell activity, and facilitate antigen release and presentation [31,32,33]. These observations provide biological plausibility for combining paclitaxel with PD-1 blockade.

However, these mechanisms should not be overinterpreted. Much of the supporting evidence derives from preclinical models or paclitaxel exposures that do not directly reproduce clinical weekly dosing, and no randomized study has demonstrated that weekly paclitaxel is immunologically superior to other chemotherapy schedules when combined with an ICI [31,32,33]. Thus, weekly paclitaxel provided KEYNOTE-B96 with an active chemotherapy backbone, whereas the contribution of its potential immunomodulatory effects to the trial’s success remains uncertain.

### 4.3. PD-L1 Status and Trial Applicability

Two features of KEYNOTE-B96 are particularly relevant when translating its results into clinical practice.

First, PD-L1 CPS is clinically relevant but should not be interpreted as showing uniformly greater relative benefit across endpoints. KEYNOTE-B96 enrolled patients irrespective of PD-L1 expression. In the CPS ≥ 1 population, the OS hazard ratio was more favorable than in the ITT population (0.76 vs. 0.82), whereas the PFS hazard ratio was not (0.72 vs. 0.70) [7]. The FDA and European Union subsequently limited the approved ovarian-cancer indication to tumors with CPS ≥ 1. Thus, CPS ≥ 1 is important for current regulatory eligibility in those jurisdictions and may enrich for OS benefit, but the trial does not support the broader statement that every efficacy endpoint showed greater benefit in the CPS ≥ 1 population.

Second, the trial population should be respected when applying the results. KEYNOTE-B96 enrolled patients with limited prior systemic treatment, whereas MIRASOL and ROSELLA also included more heavily pretreated patients [7,8,9,10]. These differences are relevant to trial applicability but should not be interpreted as evidence that the number of previous regimens is itself a dominant biological determinant of treatment choice.

Accordingly, KEYNOTE-B96 provides the most direct evidence for patients who resemble its study population and remain suitable for weekly paclitaxel. Extrapolation beyond that population should be cautious, but treatment sequencing should be driven primarily by biomarker-defined opportunities, toxicity, treatment burden, and preservation of subsequent options rather than by small differences in the number of previous treatment lines.

### 4.4. Interpreting KEYNOTE-B96 in Light of JAVELIN Ovarian 200

The contrast with JAVELIN Ovarian 200 is particularly informative because it demonstrates that successful chemoimmunotherapy cannot be explained simply by combining an ICI with chemotherapy. Importantly, PLD had been considered a particularly attractive partner for immune checkpoint blockade. In addition to being an established active treatment for PROC, anthracyclines such as doxorubicin can induce immunogenic cell death, enhance tumor-antigen presentation, and promote immune priming, providing a strong biological rationale for combining PLD with PD-1/PD-L1 blockade [25].

JAVELIN Ovarian 200 therefore provided an important test of this hypothesis, comparing avelumab monotherapy, avelumab plus PLD, and PLD alone [25]. Despite the compelling rationale, neither avelumab-containing strategy significantly improved PFS or OS. KEYNOTE-B96, in contrast, demonstrated significant improvements in both endpoints [7].

Several differences may have contributed, including the chemotherapy backbone, checkpoint inhibitor, treatment schedule, patient population, biomarker strategy, and bevacizumab use [7,25]. Weekly paclitaxel has substantial independent activity in PROC and potentially favorable immunomodulatory properties, while KEYNOTE-B96 prospectively evaluated efficacy according to PD-L1 CPS. However, no evidence establishes that any single factor explains the divergent results.

An intrinsic difference between checkpoint inhibitors also cannot be excluded. Pembrolizumab targets PD-1, whereas avelumab targets PD-L1, and differences in checkpoint target or other pharmacologic properties could theoretically contribute to different outcomes. However, the ovarian-cancer monotherapy studies do not demonstrate clearly greater intrinsic activity of pembrolizumab than other PD-1/PD-L1 inhibitors, and no head-to-head comparison has established that PD-1 rather than PD-L1 blockade explains the divergent phase III results [20,21,22,25]. This possibility therefore remains unproven.

JAVELIN Ovarian 200 therefore cautions against inferring clinical synergy from biological plausibility alone. PLD had a credible immunologic rationale, yet adding avelumab did not improve PFS or OS [25]. The contrast with KEYNOTE-B96 may reflect several differences between the studies, and the available data do not identify any single factor as the explanation.

### 4.5. Clinical Implications of KEYNOTE-B96

The patient most closely represented by KEYNOTE-B96 is one with PROC, particularly PD-L1 CPS ≥ 1 disease, who remains suitable for weekly paclitaxel and has no major contraindication to immune checkpoint inhibition [7].

However, eligibility for KEYNOTE-B96 does not necessarily mean that it should always be used first. An FRα-high patient may also be eligible for MIRV; HER2 expression may provide an additional ADC option; and relacorilant plus nab-paclitaxel provides a biomarker-unselected phase III strategy [8,9,10,11]. Pre-existing peripheral neuropathy may further alter the relative attractiveness of taxane-containing regimens.

Thus, the success of KEYNOTE-B96 creates a new clinical decision rather than eliminating one: in whom should chemoimmunotherapy be used now, and in whom should another active strategy be selected first? This question requires comparison of efficacy, biomarker eligibility, toxicity, and treatment burden across the major therapeutic options considered below.

## 5. Major Evidence-Supported and Biomarker-Directed Strategies in Platinum-Resistant Ovarian Cancer

### 5.1. Parallel Treatment Strategies Now Shape the PROC Landscape

The pivotal studies differed in biomarker selection, prior treatment requirements, and trial design, as summarized in Table 2. KEYNOTE-B96 was a biomarker-unselected PROC trial with prespecified PD-L1-defined analyses; MIRASOL was restricted to FRα-high PROC; ROSELLA required prior bevacizumab but no tumor biomarker; and DESTINY-PanTumor02 was a phase II basket trial of previously treated HER2-expressing solid tumors rather than an ovarian cancer-specific randomized trial [7,8,9,10,11]. These differences should guide interpretation and preclude simple cross-trial ranking. T-DXd has HER2-directed solid-tumor approvals based on HER2 status rather than an ovarian cancer-specific indication in several major markets, but its ovarian-cancer evidence remains nonrandomized and is not specific to PROC [11].

### 5.2. MIRASOL: Phase III Evidence for FRα-High Disease

MIRASOL randomized 453 patients with FRα-high PROC to MIRV or investigator’s-choice chemotherapy with weekly paclitaxel, pegylated liposomal doxorubicin, or topotecan [8]. FRα-high expression was defined using the PS2+ scoring method as ≥ 75% of viable tumor cells showing moderate (2+) or strong (3+) membranous FRα staining. In the primary analysis, median PFS was 5.6 versus 4.0 months (HR 0.65, 95% CI 0.52–0.81; *p* < 0.001), ORR was 42.3% versus 15.9%, and median OS was 16.5 versus 12.8 months (HR 0.67, 95% CI 0.50–0.89; *p* = 0.005), favoring MIRV [8]. With longer follow-up (median 30.5 months), the treatment effect remained consistent: median PFS was 5.6 versus 4.0 months (HR 0.63, 95% CI 0.51–0.79), ORR was 41.9% versus 15.9%, and median OS was 16.9 versus 13.3 months (HR 0.68, 95% CI 0.53–0.84; descriptive *p* = 0.0004) [9].

In the final MIRASOL safety analysis, grade ≥ 3 treatment-emergent adverse events (TEAEs) occurred in 44% versus 55% of patients, serious adverse events in 25% versus 33%, and TEAEs leading to treatment discontinuation in 11% versus 15% with MIRV and chemotherapy, respectively [9]. In the primary analysis, treatment-related deaths occurred in one patient (0.5%) in each group (neutropenic sepsis with MIRV and septic shock with topotecan) [8]. The toxicity profiles differed qualitatively. Ocular adverse events were characteristic of MIRV, occurring in 56.0% of patients; grade 3 blurred vision and keratopathy occurred in 7.8% and 9.2%, respectively, and nearly all ocular events resolved to grade 0 or 1 [8]. Peripheral neuropathy occurred in both groups (21.6% with MIRV vs. 14.5% with chemotherapy) and was predominantly low grade, whereas hematologic toxicities such as neutropenia and anemia were more frequent with chemotherapy [8]. Longer follow-up identified no new safety signals [9]. Overall, MIRV reduced several measures of severe toxicity relative to investigator-choice chemotherapy, while introducing an ocular-toxicity profile that requires structured prevention and surveillance.

### 5.3. ROSELLA: A Biomarker-Unselected Phase III Strategy

ROSELLA tested glucocorticoid receptor antagonism as a strategy to enhance taxane efficacy without tumor-biomarker selection [10]. The phase III trial randomized 381 patients with PROC and prior bevacizumab to relacorilant plus nab-paclitaxel or nab-paclitaxel alone. Median PFS was 6.5 versus 5.5 months (HR 0.70, 95% CI 0.54–0.91; *p* = 0.0076), ORR was 36.9% versus 30.1%, and final median OS was 16.0 versus 11.9 months (HR 0.65, 95% CI 0.51–0.83; *p* = 0.0004) [10].

The rationale for GR antagonism is supported by translational and preclinical work. In ovarian-cancer models, glucocorticoid receptor activation increases expression of the pro-survival genes SGK1 and DUSP1/MKP1 and can reduce chemotherapy-induced apoptosis; related patient-tissue data show induction of these genes after dexamethasone exposure [34]. Relacorilant and other GR antagonists have shown reversal of glucocorticoid-mediated chemoprotection in preclinical systems, as summarized in a recent GR-focused review [35]. These observations provide the biological rationale for combining GR antagonism with taxane therapy; they do not establish clinical efficacy. The PFS and OS benefit of relacorilant plus nab-paclitaxel is supported independently by the randomized phase III ROSELLA trial [10].

Grade ≥ 3 adverse events occurred in 74.5% of patients receiving relacorilant plus nab-paclitaxel and 59.5% receiving nab-paclitaxel alone, and serious adverse events occurred in 35.1% and 23.7%, respectively [10]. The most common toxicities, including neutropenia, anemia, fatigue, nausea, and peripheral neuropathy, were largely consistent with the nab-paclitaxel backbone; peripheral neuropathy occurred at comparable frequency in the two groups. Because treatment exposure was longer with the combination, crude event rates should be interpreted cautiously: adverse-event rates were similar between groups after adjustment for treatment duration, and no new safety signal was identified with additional follow-up [10]. No fatal adverse events were attributed specifically to relacorilant in the final follow-up report.

ROSELLA also shows why response rate alone is an incomplete measure of treatment value: the ORR difference was modest despite significant PFS and OS effects [10]. The regimen does not require a tumor biomarker, but it is not universally suitable because it remains taxane-based and, in the pivotal trial and U.S. indication, prior bevacizumab was required.

### 5.4. HER2-Directed ADCs: Substantial Activity, but a Different Level of Evidence

In DESTINY-PanTumor02, trastuzumab deruxtecan (T-DXd) was evaluated in a phase II multicohort single-arm study of previously treated HER2-expressing solid tumors [11]. The ovarian cancer cohort included 40 patients and was not restricted specifically to PROC. ORR was 45.0% (95% CI 29.3–61.5) overall, 63.6% among 11 patients with centrally confirmed HER2 IHC 3+ tumors, and 36.8% among 19 patients with centrally confirmed HER2 IHC 2+ tumors. Median PFS and OS in the overall ovarian cohort were 5.9 and 13.2 months, respectively; corresponding estimates in the small IHC 3+ subgroup were 12.5 and 20.0 months [11]. The ovarian cohort was heavily pretreated: 72.5% had received prior anti-VEGF therapy, 37.5% prior PARP inhibitor therapy, and 82.5% had platinum-resistant disease [11].

These findings demonstrate clinically meaningful activity, particularly in HER2 IHC 3+ tumors, but should not be directly compared with randomized phase III PROC results. In the 2026 analysis of 120 patients across the ovarian, endometrial, and cervical cohorts, adjudicated interstitial lung disease/pneumonitis occurred in 13 patients (10.8%), including one grade 5 event [11]. HER2 positivity therefore provides an additional therapeutic opportunity, but current evidence does not establish the optimal position of T-DXd relative to phase III-established PROC strategies. Regulatory criteria differ by region. As of the 22 September 2026 cut-off, HER2-directed solid-tumor indications are available in the United States and European Union for tumors meeting their respective HER2 criteria; in Japan, T-DXd is approved for HER2-positive advanced or recurrent solid tumors, with HER2 positivity defined as HER2 (ERBB2) gene amplification or HER2 overexpression by IHC 3+, when standard treatment is difficult [36,37,38].

## 6. A Clinically Actionable Framework for PROC Treatment

### 6.1. Start with All Evidence-Supported Options, Not Only the Newest Regimens

At each decision point, the first step is to identify all treatments that are both evidence-supported and realistically deliverable. Established options remain important: weekly paclitaxel, PLD, topotecan, or gemcitabine, with bevacizumab when clinically appropriate, continue to be used in PROC [1,2,3,4]. Oral etoposide and metronomic oral cyclophosphamide-based therapy can be useful in selected patients when intravenous therapy, frequent visits, toxicity, access, or cost make other regimens impractical [5,6]. The newer strategies reviewed here should therefore be integrated into, rather than substituted for, the conventional therapeutic landscape.

### 6.2. Choosing Among Simultaneously Applicable Options

When more than one option is available, the decision should be driven by the patient’s current clinical constraints rather than by cross-trial ranking. Table 2 and Table 3 make the practical differences between the newer strategies explicit: Table 2 shows biomarker/eligibility requirements and administration schedules, whereas Table 3 summarizes regulatory context and treatment burden. Baseline neuropathy, marrow reserve, ocular disease, pulmonary history, need for systemic corticosteroids, prior bevacizumab, biomarker status, and patient preference can therefore change which option is most appropriate even when several are technically eligible [7,8,9,10,11]. Table 4 translates these considerations into an operational clinical framework. It identifies evidence-supported options for common decision points and separates factors favoring consideration from factors that limit use. Treatments are not ranked because no head-to-head or prospective sequencing trial establishes a preferred order among the newer phase III-supported strategies.

### 6.3. Peripheral Neuropathy as a Determinant of Treatment Sequencing

Peripheral neuropathy is particularly relevant to sequencing in PROC because many patients have residual neurotoxicity from previous platinum-taxane therapy. For weekly paclitaxel, an ovarian-cancer Gynecologic Oncology Group phase II study using 80 mg/m^2^ weekly reported grade 2 neuropathy in 21% and grade 3 neuropathy in 4% of patients [28]; another platinum/paclitaxel-refractory phase II study discontinued treatment for neuropathy in 4 of 52 toxicity-evaluable patients [29]. In ROSELLA, peripheral neuropathy of any grade was reported in 19.1% of patients receiving relacorilant plus nab-paclitaxel and 17.4% receiving nab-paclitaxel alone [10]. Grade ≥ 3 peripheral neuropathy was not separately reported in the published phase III safety table, and therefore a grade ≥ 3 rate should not be inferred from the overall incidence. The combination used nab-paclitaxel 80 mg/m^2^ on days 1, 8, and 15 of each 28-day cycle, whereas the monotherapy arm used 100 mg/m^2^ on the same schedule [10]. With MIRV in MIRASOL, peripheral neuropathy of any grade occurred in 21.6% of patients, with grade ≥ 3 events in 1.4% [8]. Thus, where severity-specific data are available, grade ≥ 3 neuropathy was 4% in the earlier weekly-paclitaxel phase II study and 1.4% with MIRV in MIRASOL, whereas ROSELLA reported only the overall peripheral-neuropathy frequencies in its published phase III safety summary. These figures are presented to define the toxicity observed with each strategy, not to rank them. The studies differed in population, prior taxane exposure, dose and schedule, treatment duration, adverse-event definitions, and reporting; the weekly-paclitaxel data are from earlier phase II studies, whereas MIRASOL and ROSELLA were contemporary randomized trials. Accordingly, no valid numerical comparison of CIPN risk can be made across these studies. Clinically, however, additional taxane exposure remains an important concern when residual CIPN is functionally significant. In FRα-high disease, MIRV offers a non-taxane option when further taxane exposure is undesirable, while recognizing that MIRV itself can cause neuropathy. Neuropathy should therefore be treated as a regimen-specific and patient-specific constraint rather than as a basis for cross-trial toxicity ranking.

### 6.4. Treatment Burden, Financial Accessibility, and Later-Line Attrition

Treatment burden includes more than adverse-event frequency. In this review, it refers to infusion and visit frequency, route of administration, required laboratory or specialty monitoring, time spent receiving or recovering from treatment, caregiver demands, transportation, employment disruption, and out-of-pocket or other financial costs [49,50]. Table 2 summarizes route and administration schedule, while Table 3 highlights the principal monitoring and practical requirements. These differences can be clinically important for patients who live far from treatment centers or have substantial work, family, or caregiver responsibilities. Regimen-specific toxicity management should also account for established guidance on immune-related adverse events [51].

Financial burden can also determine whether an otherwise appropriate treatment is feasible. Drug acquisition costs, insurance coverage, reimbursement, and patient out-of-pocket expenses differ markedly among health-care systems and change over time; indirect costs also rise with frequent visits and monitoring [49]. For that reason, direct numerical price comparisons across the United States, Europe, and Japan would be rapidly outdated and potentially misleading. Financial accessibility should nevertheless be discussed explicitly during shared decision-making, and a less resource-intensive established regimen may be reasonable when access to a newer treatment is limited.

The concept of keeping mechanistically distinct treatments available for later lines also requires caution. No prospective study has shown that delaying an active therapy preserves its efficacy or improves overall outcome. Moreover, attrition occurs between treatment lines: progression, declining performance status, organ dysfunction, bowel obstruction, cumulative toxicity, or patient preference may prevent delivery of a treatment reserved for later. Thus, sequencing should balance the expected value of the current treatment against the probability that subsequent therapy will actually remain feasible, rather than deferring an active regimen solely to preserve it.

### 6.5. What Is Known After MIRV or Taxane-Based Phase III Therapy?

Evidence for treatment after the newer phase III strategies remains limited. After MIRV, taxane activity is not necessarily lost because MIRV delivers DM4 through FRα-mediated internalization whereas paclitaxel and nab-paclitaxel are systemically delivered taxanes. In a retrospective series of 62 patients who received both MIRV and weekly paclitaxel, treatment order did not significantly alter response to either agent [48]. The study is hypothesis-generating and does not establish an optimal sequence, but it supports considering taxane-based therapy after MIRV when neuropathy and other clinical factors permit.

After KEYNOTE-B96 or relacorilant plus nab-paclitaxel, biomarker-directed treatment with MIRV or T-DXd may be appropriate when the corresponding target and regulatory criteria are met. Conventional non-platinum chemotherapy with or without bevacizumab and clinical trials also remain relevant after any of the newer regimens. Conversely, another taxane-based strategy may be difficult after substantial cumulative neuropathy. These post-progression choices are best viewed as conditional options, not as a fixed sequence.

### 6.6. Histologic Subtype as a Modifier of Biomarker-Directed Treatment Selection

Histologic subtype may inform biomarker testing but should not substitute for direct biomarker assessment. FRα-high expression is generally enriched in serous carcinomas, although reported prevalence varies across studies: approximately 44–47% in HGSC, 25–43% in LGSC, 1–35% in clear-cell, 2–29% in endometrioid, and 0–7% in mucinous carcinomas [52,53,54]. The particularly wide variation among non-serous histotypes highlights the influence of assay methodology, sampling, and cohort composition and argues against using histology to determine whether FRα testing should be performed. PD-L1 expression also varies by histotype and assay, with CPS positivity reported in 27.5–41.5% of HGSC and 27.3–29.6% of clear-cell carcinomas, depending on the antibody clone [55]. HER2 IHC 2+/3+ expression was observed in 23.6% of ovarian cancers overall and was particularly frequent in mucinous (50.0%) and clear-cell (51.2%) carcinomas [56]. However, biomarker prevalence should not be equated with histology-specific therapeutic efficacy; preliminary retrospective data suggest that HER2 expression may not predict uniform T-DXd activity across ovarian cancer histotypes [57].

Histologic eligibility also differed among the pivotal trials (Table 2), but the available evidence base was predominantly derived from HGSC. KEYNOTE-B96 permitted broader epithelial histologies, yet 86.0% of patients had HGSC; MIRASOL was restricted to HGSC (100%); ROSELLA permitted only high-grade serous, high-grade endometrioid, and carcinosarcoma histologies; and DESTINY-PanTumor02 did not restrict ovarian cancer enrollment by histologic subtype, but its ovarian cancer cohort comprised only 40 patients. Thus, broader eligibility should not be interpreted as established efficacy across histologic subtypes, and current evidence is insufficient to define an optimal treatment strategy for non-HGSC ovarian cancers as a group or for individual non-HGSC histotypes. Histology should therefore be used to inform biomarker testing and the strength of evidence supporting treatment extrapolation, rather than as an independent determinant of treatment selection.

## 7. Future Directions

### 7.1. Establishing Evidence for Longitudinal Treatment Sequencing

Current sequencing strategies in PROC are based largely on trial applicability, biomarker eligibility, toxicity, and clinical judgment rather than direct comparative evidence. Prospective studies should evaluate treatment sequences rather than individual therapies in isolation, particularly when patients are eligible for more than one active strategy.

Studies comparing sequences such as MIRV followed by KEYNOTE-B96 versus the reverse should assess not only first-treatment PFS but also PFS2, feasibility of subsequent therapy, cumulative toxicity and treatment burden, quality of life, and OS. Prospective registries may complement randomized sequencing trials. Longitudinal reassessment of biomarkers, including repeat tissue sampling or validated liquid-biopsy approaches, should also be explored because target expression and resistance mechanisms may evolve during treatment.

### 7.2. Beyond PD-L1: Multidimensional Biomarkers for Immunotherapy

With phase III evidence now supporting chemoimmunotherapy in PROC, improved biomarkers for immunotherapy are an important research priority. PD-L1 CPS provides clinically useful enrichment, but is unlikely to capture the biological complexity underlying response to PD-1 blockade [7].

Potential complementary biomarkers include CD8+ T-cell infiltration, the CD8+/Treg ratio, immunosuppressive myeloid populations, immune gene-expression profiles, tumor mutational burden, metabolic features, and systemic inflammatory markers [12,13,14,15,16,58,59,60,61]. However, ovarian cancer-specific evidence remains inconsistent. In exploratory KEYNOTE-100 analyses, neither T-cell-inflamed gene-expression profile nor continuous tumor mutational burden clearly predicted pembrolizumab outcomes, whereas higher tumor-compartment densities of CD11c+ and CD11c+/MHCII−/CD163−/CD68− myeloid-cell phenotypes were positively associated with objective response; glycolysis- and hypoxia-related gene-expression signatures showed negative associations with selected longer-term outcomes [59]. These findings remain exploratory and are not validated treatment-selection biomarkers.

Future biomarker development should therefore move beyond identifying patients who are simply eligible for pembrolizumab-based therapy toward identifying those most likely to derive meaningful incremental benefit from adding pembrolizumab to chemotherapy. Such biomarkers may ultimately need to support both treatment selection and longitudinal sequencing.

## 8. Conclusions

PROC now includes several evidence-supported and biomarker-defined treatment opportunities in addition to established non-platinum chemotherapy. Their evidence bases are not interchangeable: positive phase III results, regulatory approval, guideline incorporation, and routine availability are distinct steps, and each can differ across jurisdictions. No prospective study has established an optimal sequence among KEYNOTE-B96, MIRV, relacorilant plus nab-paclitaxel, T-DXd, and conventional regimens. The framework proposed here is therefore an evidence-informed clinical approach rather than a validated treatment algorithm. In practice, the choice at each line should weigh the expected benefit of treatment now against biomarker eligibility, prior exposure, residual toxicity, monitoring and financial burden, patient priorities, and the probability that subsequent therapy will remain feasible. Prospective sequencing studies are needed to determine whether particular treatment orders improve cumulative clinical benefit.

An important implication is that maintaining future options should not become a reason to postpone an active treatment for a patient who is eligible for it now. Later-line attrition is clinically relevant in PROC, and some patients will not remain fit enough to receive a treatment reserved for the future. Future trials should therefore evaluate longitudinal outcomes, including PFS2, treatment attrition, cumulative toxicity and burden, quality of life, and overall survival.

## Figures and Tables

**Table 1:** Evolution of immunotherapy for platinum-resistant ovarian cancer: from monotherapy to phase III chemoimmunotherapy.

Trial	Phase/Design	Treatment	Primary Endpoint	Key Efficacy Findings	Clinical Interpretation	Contribution to Subsequent Development
KEYNOTE-028	Phase Ib, single-arm	Pembrolizumab (anti-PD-1 antibody)	ORR	ORR 11.5% Median PFS 1.9 mo Median OS 13.8 mo	Demonstrated initial clinical activity of PD-1 blockade in ovarian cancer	Established proof-of-concept for immune checkpoint inhibition [20]
KEYNOTE-100	Phase II, single-arm	Pembrolizumab (anti-PD-1 antibody)	ORR	ORR 8.0% overall; higher ORR in PD-L1-positive tumors Median PFS 2.1 mo Median OS 18.7 mo	PD-L1 enriched for response but remained an imperfect predictive biomarker	Supported biomarker-based patient selection [21]
NINJA	Phase III, randomized	Nivolumab (anti-PD-1 antibody) Investigator’s-choice chemotherapy	OS	ORR 7.6% vs. 13.2% Median PFS 2.0 vs. 3.8 mo Median OS 10.1 vs. 12.1 mo	Immune checkpoint inhibitor monotherapy was inferior to chemotherapy	Demonstrated that PD-1 blockade alone is insufficient in PROC [22]
TOPACIO/KEYNOTE-162	Phase I/II, single-arm	Niraparib (PARP inhibitor) + pembrolizumab	ORR	ORR 18% Disease control rate 65% Median PFS 3.4 mo	PARP inhibition may augment antitumor immunity	Supported biologically rational combination strategies [23]
NRG-GY003	Phase II, randomized	Nivolumab (anti-PD-1 antibody) Nivolumab + ipilimumab (anti-CTLA-4 antibody)	ORR	ORR 12.2% vs. 31.4% Median PFS 2.0 vs. 3.9 mo Median OS 21.8 vs. 28.1 mo	Dual checkpoint blockade produced greater antitumor activity	Demonstrated that immune activation could be enhanced through combination immunotherapy [24]
JAVELIN Ovarian 200	Phase III, randomized	Avelumab (anti-PD-L1) Avelumab + PLD PLD	PFS and OS	ORR 13.3%, 18.5%, and 4.2% No significant improvement in PFS or OS for either avelumab-containing arm	Not all chemotherapy backbones enhance immune checkpoint inhibition	Highlighted the importance of selecting an appropriate chemotherapy partner [25]
KEYNOTE-B96	Phase III, randomized, double-blind	Pembrolizumab + weekly paclitaxel ± bevacizumab Placebo + weekly paclitaxel ± bevacizumab	PFS	Median PFS 8.3 vs. 6.4 mo (HR 0.70) Median OS 17.7 vs. 14.0 mo (HR 0.82) ORR 50.4% vs. 40.8%	First phase III chemoimmunotherapy trial to improve PFS and OS in PROC; benefit enriched by PD-L1 CPS	Established chemoimmunotherapy as a new option while highlighting the need to balance efficacy, toxicity, and treatment burden [7]

CPS, combined positive score; CTLA-4, cytotoxic T-lymphocyte-associated protein 4; HR, hazard ratio; ORR, objective response rate; OS, overall survival; PARP, poly(ADP-ribose) polymerase; PD-1, programmed cell death protein 1; PD-L1, programmed death-ligand 1; PFS, progression-free survival; PLD, pegylated liposomal doxorubicin; PROC, platinum-resistant ovarian cancer.

**Table 2:** Comparison of pivotal clinical trials establishing major treatment strategies for platinum-resistant ovarian cancer.

Characteristic	KEYNOTE-B96 [7]	MIRASOL [8,9]	ROSELLA [10]	DESTINY-PanTumor02 [11]
Treatment	Pembrolizumab (anti-PD-1 antibody) + weekly paclitaxel ± bevacizumab vs. placebo + weekly paclitaxel ± bevacizumab	Mirvetuximab soravtansine (FRα-directed ADC) vs. investigator’s-choice chemotherapy (weekly paclitaxel, PLD, or topotecan)	Relacorilant (selective GR antagonist) + nab-paclitaxel vs. nab-paclitaxel	Trastuzumab deruxtecan (HER2-directed ADC; single-arm)
Route and schedule	Pembrolizumab 400 mg IV q6w + paclitaxel 80 mg/m^2^ IV D1, 8, 15 q21d ± bevacizumab 10 mg/kg IV q2w.	6 mg/kg adjusted ideal body weight (AIBW) IV q3w.	Relacorilant 150 mg PO day before/of/after each infusion + nab-paclitaxel 80 mg/m^2^ IV D1, 8, 15 q28d.	5.4 mg/kg IV q3w.
Phase/design	Phase III, randomized, double-blind	Phase III, randomized, open-label	Phase III, randomized, open-label	Phase II, multicohort, open-label, single-arm
Enrolled population	Biomarker-unselected PROC; 1–2 prior systemic regimens	FRα-high PROC; 1–3 prior systemic regimens	Biomarker-unselected PROC; 1–3 prior systemic regimens; prior bevacizumab required	Previously treated HER2-expressing (IHC 2+/3+) advanced solid tumors; ovarian cancer cohort (*n* = 40), not restricted to PROC
Histologic eligibility/enrolled population	Broad epithelial histologies permitted, but the enrolled population remained predominantly HGSC (86.0%); clear-cell carcinoma accounted for 7.8%, and other non-HGSC subtypes were uncommon.	High-grade serous carcinoma only (100%).	High-grade serous, high-grade endometrioid, or carcinosarcoma with ≥30% epithelial component were eligible; the published supplementary baseline table does not report the enrolled histologic distribution.	Ovarian cancer enrollment was not restricted to a single histotype; however, the ovarian cohort was small (*n* = 40), and histology-specific efficacy was not established.
Other relevant prior treatment	Prior bevacizumab 46%; prior PARPi 39%	Prior bevacizumab 62%; prior PARPi 55%	Prior bevacizumab 100%; prior PARPi 61%	Prior anti-vascular endothelial growth factor (VEGF) therapy 72.5%; prior PARPi 37.5%; 82.5% had platinum-resistant disease.
Primary endpoint(s)	PFS (ITT and PD-L1 CPS ≥ 1 populations)	PFS	PFS and OS	ORR
Median PFS	8.3 vs. 6.4 mo	5.6 vs. 4.0 mo (final follow-up)	6.5 vs. 5.5 mo	5.9 mo (ovarian cohort)
PFS HR (95% CI); *p* value	0.70 (0.58–0.84); *p* < 0.0001	Primary analysis: 0.65 (0.52–0.81); *p* < 0.001. Final follow-up: 0.63 (0.51–0.79); final *p* value not reported.	0.70 (0.54–0.91); *p* = 0.0076	—
Median OS	17.7 vs. 14.0 mo	16.9 vs. 13.3 mo (final follow-up)	16.0 vs. 11.9 mo (final OS analysis)	13.2 mo (ovarian cohort)
OS HR (95% CI); *p* value	0.82 (0.69–0.97); *p* = 0.011	0.68 (0.53–0.84); descriptive *p* = 0.0004	0.65 (0.51–0.83); *p* = 0.0004	—
ORR	50.4% vs. 40.8%	41.9% vs. 15.9% (final follow-up)	36.9% vs. 30.1%	45.0% (ovarian cohort)
Biomarker/eligibility and subgroup findings	PD-L1 CPS ≥ 1: PFS 8.3 vs. 7.2 mo (HR 0.72); OS 18.2 vs. 14.0 mo (HR 0.76); ORR 53.0% vs. 46.6%	Entire trial restricted to FRα-high disease	No validated predictive biomarker identified	Ovarian cohort by central HER2 testing: ORR 63.6% in IHC 3+ (*n* = 11) vs. 36.8% in IHC 2+ (*n* = 19)
Selected safety findings	Grade ≥ 3 treatment-related AEs 68% vs. 55%; key practical toxicities include paclitaxel-related neuropathy/myelosuppression and pembrolizumab-related immune AEs.	Grade ≥ 3 TEAEs: 44% vs. 55% (final follow-up); characteristic MIRV toxicities include ocular AEs and peripheral neuropathy.	Grade ≥ 3 AEs 74.5% vs. 59.5%; peripheral neuropathy of any grade 19.1% vs. 17.4% (grade ≥ 3 neuropathy not separately reported); other important toxicities are predominantly taxane-related, including myelosuppression.	ILD/pneumonitis is the key serious toxicity; in the 120-patient gynecologic-cancer analysis, adjudicated ILD/pneumonitis occurred in 10.8%.

Note: Histologic eligibility differed among trials, but the available evidence base was predominantly derived from HGSC. Broader eligibility should not be interpreted as established efficacy within individual non-HGSC histotypes, which were generally represented by small patient numbers. Note: Safety endpoints are reported according to the definitions used in the respective trials and should not be directly compared across studies. For MIRASOL, efficacy estimates include both the primary and final follow-up analyses, whereas the safety data presented in this table are from the final follow-up analysis [9]. For DESTINY-PanTumor02, the prior-treatment percentages shown are specific to the ovarian cohort, whereas the ILD/pneumonitis estimate is from the 120-patient gynecologic-cancer analysis. ADC, antibody–drug conjugate; AE, adverse event; AIBW, adjusted ideal body weight; CI, confidence interval; CPS, combined positive score; D1, day 1; D8, day 8; D15, day 15; FRα, folate receptor alpha; GR, glucocorticoid receptor; HER2, human epidermal growth factor receptor 2; HGSC, high-grade serous carcinoma; HR, hazard ratio; IHC, immunohistochemistry; ILD, interstitial lung disease; ITT, intention-to-treat; IV, intravenous; ORR, objective response rate; OS, overall survival; PARPi, poly(ADP-ribose) polymerase inhibitor; PD-1, programmed cell death protein 1; PD-L1, programmed death-ligand 1; PFS, progression-free survival; PLD, pegylated liposomal doxorubicin; PO, oral; PROC, platinum-resistant ovarian cancer, q2w, every 2 weeks; q3w, every 3 weeks; q6w, every 6 weeks; q21d, every 21 days; q28d, every 28 days; VEGF, vascular endothelial growth factor.

**Table 3:** Regulatory status, guideline/routine-practice context, and treatment burden of major newer strategies for platinum-resistant ovarian cancer as of 22 September 2026.

Strategy/Pivotal Study (Key Refs)	Regulatory Status (Cut-Off: 22 September 2026)	Guideline/Routine-Practice Context	Treatment Burden/Practical Considerations
Pembrolizumab + weekly paclitaxel ± bevacizumab (KEYNOTE-B96) [7,39,40,41]	US: FDA approved for PD-L1 CPS ≥ 1 PROC after 1–2 prior systemic regimens. EU: EC approved for PD-L1 CPS ≥ 1 PROC after 1–2 prior systemic regimens. Japan: approved 16 September 2026 for platinum-resistant recurrent ovarian cancer; the Japanese approval announcement does not state a PD-L1 restriction.	Positive randomized phase III evidence; PD-L1 CPS ≥ 1 is required for the US and EU indications.	Weekly paclitaxel infusion visits; pembrolizumab q6w; additional q2w bevacizumab visits when used. Monitor neuropathy/myelosuppression, immune-related AEs, and blood pressure/proteinuria with bevacizumab.
Mirvetuximab soravtansine (MIRASOL) [8,9,42,43,44,45]	US: FDA full approval for FRα-positive PROC after 1–3 prior regimens. EU: marketing authorization for FRα-positive platinum-resistant high-grade serous disease after 1–3 prior regimens. Japan: approved 16 September 2026 for FRα-positive platinum-resistant recurrent ovarian cancer.	Positive randomized phase III evidence; recommended in the 2026 ESMO update for FRα-high recurrent disease after 1–3 prior therapies.	q3w infusion. Prophylactic eye care and scheduled ophthalmic assessment are required; ocular toxicity may require dose modification. Monitor peripheral neuropathy.
Relacorilant + nab-paclitaxel (ROSELLA) [10,46,47]	US: FDA approved after 1–3 prior regimens; prior bevacizumab required. EU: CHMP positive opinion 18 September 2026; EC marketing authorization not yet issued at cut-off. Japan: no approval identified at cut-off.	Positive randomized phase III evidence in a population with prior bevacizumab; no biomarker selection required.	Nab-paclitaxel IV on D1, D8, and D15 q28d; relacorilant PO on the day before, day of, and day after each infusion. Monitor myelosuppression and cumulative taxane-related neuropathy.
Trastuzumab deruxtecan (DESTINY-PanTumor02) [11,36,37,38]	US: FDA accelerated approval for HER2 IHC 3+ unresectable/metastatic solid tumors after prior systemic treatment when no satisfactory alternatives exist. EU: approval for HER2 IHC 3+ solid tumors after prior treatment when no satisfactory options exist. Japan: approved for HER2-positive advanced/recurrent solid tumors refractory or intolerant to standard treatment (IHC 3+ or HER2 gene amplification).	HER2-directed solid-tumor indications; ovarian evidence is single-arm rather than randomized phase III evidence.	q3w infusion; HER2 testing required. Monitor closely for ILD/pneumonitis; cardiac monitoring as clinically appropriate.

Abbreviations: AE, adverse event; CHMP, Committee for Medicinal Products for Human Use; CPS, combined positive score; D1, day 1; D8, day 8; D15, day 15; EC, European Commission; ESMO, European Society for Medical Oncology; EU, European Union; FDA, US Food and Drug Administration; FRα, folate receptor alpha; HER2, human epidermal growth factor receptor 2; IHC, immunohistochemistry; ILD, interstitial lung disease; IV, intravenous; PD-L1, programmed death-ligand 1; PO, oral; PROC, platinum-resistant ovarian cancer; q2w, every 2 weeks; q28d, every 28 days; q3w, every 3 weeks; q6w, every 6 weeks. Regulatory approval does not necessarily indicate immediate routine availability; guideline incorporation, reimbursement, and access to required biomarker or companion-diagnostic testing may vary across jurisdictions and may lag regulatory approval.

**Table 4:** Clinical decision framework for treatment selection and sequencing in platinum-resistant ovarian cancer.

Clinical Situation	Evidence-Supported Options	Factors Favoring Consideration	Factors Limiting Use/Caveats
FRα-high PROC	MIRV [8,9]; other eligible phase III or conventional options if unsuitable.	• Biomarker-selected phase III evidence • Non-taxane option when further taxane exposure is undesirable	• Ocular prophylaxis/monitoring required • Consider baseline neuropathy; MIRV-associated neuropathy is usually low grade but can require dose modification
PD-L1 CPS ≥ 1; taxane/ICI suitable	Pembrolizumab + weekly paclitaxel ± bevacizumab [7]; other biomarker-directed options when eligible.	• Phase III PFS and OS benefit • OS HR more favorable in CPS ≥ 1 than ITT	• Weekly paclitaxel may worsen residual neuropathy • Avoid ICI when clinically contraindicated • Frequent infusion visits; add bevacizumab-specific monitoring when used
Prior bevacizumab; taxane feasible	Relacorilant + nab-paclitaxel [10] where approved/available; biomarker-directed or conventional options remain alternatives.	• ROSELLA required prior bevacizumab • Phase III PFS and OS benefit • No biomarker selection	• Nab-paclitaxel D1, 8, 15 q28d • Residual/cumulative taxane neuropathy may limit use • Myelosuppression requires monitoring
HER2 IHC 3+; eligible under regional label	T-DXd [11,36,37,38]; other PROC options if unavailable or unsuitable.	• HER2-directed, non-taxane option • Strongest response signal in IHC 3+ tumors	• Ovarian cohort small (*n* = 40), single-arm, and not PROC-specific • ILD/pneumonitis risk requires active surveillance
No actionable biomarker/testing unavailable	Relacorilant + nab-paclitaxel if eligible [10]; otherwise weekly paclitaxel, PLD, topotecan, or gemcitabine ± bevacizumab [1,2,3,4]; selected oral cytotoxic therapy in later lines [5,6].	• Does not require FRα/HER2 selection • Conventional agents remain widely usable	• Conventional non-platinum chemotherapy generally has modest response/PFS • Match agent to residual toxicity: avoid taxane with troublesome neuropathy; avoid marrow-suppressive therapy with poor reserve
Clinically relevant neuropathy	Prefer non-taxane options when eligible: MIRV for FRα-high disease; T-DXd for HER2-eligible disease; or non-taxane chemotherapy ± bevacizumab.	• Avoids additional taxane exposure • MIRV is a non-taxane option in FRα-high disease	• MIRV can also cause neuropathy, but do not infer relative risk from cross-trial rates • With function-limiting chemotherapy-induced peripheral neuropathy (CIPN), favor a regimen without additional taxane exposure when feasible
ICI contraindication/preference to avoid immune toxicity	MIRV if FRα-high; relacorilant + nab-paclitaxel if eligible; T-DXd if HER2-eligible; or conventional chemotherapy ± bevacizumab.	• Avoids checkpoint-inhibitor toxicity	• Select by dominant competing toxicity: ocular toxicity (MIRV), neuropathy/myelosuppression (nab-paclitaxel), ILD risk (T-DXd), or residual toxicity from prior chemotherapy
Progression after a newer strategy	Choose among still-unused eligible classes: MIRV, pembrolizumab + weekly paclitaxel ± bevacizumab, relacorilant + nab-paclitaxel, T-DXd, conventional chemotherapy ± bevacizumab, or a clinical trial.	• Reassess biomarkers, prior exposure, and residual toxicity • Paclitaxel can retain activity after MIRV in retrospective data [48]	• Do not delay an active current-line option solely to preserve it for later • After taxane-containing therapy, residual neuropathy may favor MIRV/T-DXd/non-taxane chemotherapy when eligible
Non-HGSC histology/histology-specific evidence is limited	• Perform direct biomarker testing rather than selecting treatment from histology alone • FRα testing should not be withheld solely because a tumor is non-HGSC • Consider HER2 and PD-L1 testing when clinically relevant	• Histology can inform the pre-test probability of biomarker positivity • FRα-high is consistently common in serous tumors, but non-serous prevalence varies substantially across studies • HER2 expression may be enriched in mucinous and clear-cell carcinomas	• The pivotal evidence is HGSC-restricted, HGSC-dominant, restricted to selected high-grade histologies, or based on a small ovarian cohort; none provides sufficient comparative evidence to define an optimal treatment for non-HGSC as a group or for individual non-HGSC histotypes • Biomarker positivity does not by itself establish histology-specific efficacy • Histology should guide testing and evidence interpretation, not create a treatment hierarchy

Abbreviations: AE, adverse event; CIPN, chemotherapy-induced peripheral neuropathy; CPS, combined positive score; D1, day 1; FRα, folate receptor alpha; HER2, human epidermal growth factor receptor 2; HGSC, high-grade serous carcinoma; HR, hazard ratio; ICI, immune checkpoint inhibitor; IHC, immunohistochemistry; ILD, interstitial lung disease; ITT, intention-to-treat; MIRV, mirvetuximab soravtansine; n, number of patients; OS, overall survival; PD-L1, programmed death-ligand 1; PFS, progression-free survival; PLD, pegylated liposomal doxorubicin; PROC, platinum-resistant ovarian cancer; q28d, every 28 days; T-DXd, trastuzumab deruxtecan. Note: Options within each row are not listed in rank order. Treatment selection should reflect current eligibility, expected benefit, residual toxicity, comorbidity, patient preference, access, and the realistic feasibility of subsequent therapy.

## Data Availability

No new data were created or analyzed in this study. Data sharing is not applicable to this article.

## Abbreviations

The following abbreviations are used in this manuscript:

ADC	antibody–drug conjugate
CI	confidence interval
CPS	combined positive score
DM4	maytansinoid DM4
FRα	folate receptor alpha
HER2	human epidermal growth factor receptor 2
HR	hazard ratio
ICI	immune checkpoint inhibitor
IHC	immunohistochemistry
irAE	immune-related adverse event
ITT	intention-to-treat
MDSC	myeloid-derived suppressor cell
MIRV	mirvetuximab soravtansine
ORR	objective response rate
OS	overall survival
PARP	poly(ADP-ribose) polymerase
PD-1	programmed cell death protein 1
PD-L1	programmed death ligand 1
PFS	progression-free survival
PFS2	progression-free survival through the next line of therapy
PLD	pegylated liposomal doxorubicin
PROC	platinum-resistant ovarian cancer
T-DXd	trastuzumab deruxtecan
TIL	tumor-infiltrating lymphocyte
Treg	regulatory T cell
HGSC	high-grade serous carcinoma
LGSC	low-grade serous carcinoma
